# Cremation*Read before the Buffalo Medical Club, March 2, 1881.

**Published:** 1881-04

**Authors:** Frederick Peterson


BUFFALO

MEDICAL AND SURGICAL JOURNAL.

VOL. XX.—APRIL, 1881.—NO. 9.

### Original Communications.

### CREMATION.*

BY FREDERICK PETERSON, M. D.

CREMATION is a necessity. It will come into vogue sooner or later. It awaits the evolution of good sense from the chaos of popular sentiment. Its advocates at present are the intelligent in general and scientists in particular. Its opponents are undertakers, manufacturers of coffin fixtures, dealers in fine woods, grave diggers, florists, cemetery associations, a large number with an antique and sentimental cast of mind, and those ministers and their congregations who fear cremation may make to Omnipotence an impossibility or at least a difficulty of resurrection. The advocates increase and the opponents die. To the intelligent man, personally, it is of course of no consequence what becomes of his body after death, but only with regard to the welfare of the living.

Once men decomposed where they fell; they were the prey, as are other animals at present, of insects and wild beasts and birds. The elements which composed them formed quickly new associations, for that is nature's way. But we of later ages

* Read before the Buffalo Medical Club, March 2, 1881.

have changed the process. We allow the dead whom we have loved so much, whom we profess to revere and hold sacred, to putrefy from twelve to fifty years underground, and the richest of us rot longer in proportion to our riches, for wealth must have leaden caskets and mausoleums and vaults and sarcophagi. It is a comfort to the desolate to know that the dear possessors of beauty and goodness and glorious eyes and golden tongues and brains whose thought-secretions have made men mute, may repose luxuriously in these stony prisons for five hundred years, while the elements of once splendid structures struggle among themselves in a foul melee for escape. What do they seek? Re-union and re-employment of their forces, heat, light, electricity and mind.

From a decomposing body arise through atmosphere and rain action, carbolic acid, water, ammonia, nitrous and nitric acids, sulphuretted hydrogen, sulphuric acid, carburetted hydrogen, trimethylamin, tauryl, butyric and propionic acids, and some volatile organic matter, most of which pass off in vapor, while the mineral constituents remain in the soil to nourish what plant life there may be. These gases constantly arise from graveyards and are often distinguishable when there is overcrowding. Some excellent descriptions may be found of this in the English clergyman Haweis' cremation prelude, "Ashes to Ashes," and in Walker's "Gatherings from Graveyards." Only recently the exhalations from Greenwood have been complained of in South Brooklyn. Some of the gases, as carbonic acid, are poisonous and have a depressing effect upon the circulation, others are foul-smelling, and the volatile organic matter is sometimes so abundant as to be tasted. Putrefying animal matter introduced into the blood, by dissection for instance, has often caused a fatal pyemia. It is presumed that these noxious vapors may also give rise to toxic affections through the delicate pulmonary membranes. They do so in a concentrated form. According to Chadwick, the life of a grave-digger loses one-third of its natural duration, and often sudden death overtakes him, as in the case of three in Paris in 1852, from the inhalation of mephitic vapors. When distributed, they lower vital power. This was the report of the English Parliamentary Commissioners. Eassie says the disorders common in the vicinity of graveyards, and in fact in the neighborhood of any putrefying matter, are headache, diarrhœa, dysentery, low fevers, and ulcerated sore throat.

Naturally, it has always been among men a matter of importance to get rid of the dead. Not only were the bodies repugnant to sight and feeling, but they were detrimental to health. The modes of disposal of bodies have varied with the intelligence of the people, with the dictates of religion, with the convenience of the soil for reception, with the proximity of fuel, the sea, caves, dry air or wild animals. Consequently we have mere exposure above ground, next covering with a little earth to conceal, which is our modern inhumation, then entombment, embalming, burning, drying, sea-burial, destruction with chemicals, petrifaction and cementing. Exposure was probably practiced by the aborigines of both continents. Inhumation is the usual method now in Europe, China and America. Entombment, another mode of concealment, was established in those countries, where the nature of the land permitted it, as about Jerusalem, where there are many natural caves, which were enlarged and embellished. Embalming or mummification was practiced for many centuries in Egypt, where the peculiarly dry atmosphere and rocky caves allowed of preservation for ages. The beautiful figure of Gautier's Egyptian Tahoser endures to entrance a young Englishman after thirty centuries, and there is something in this æsthetic as well as practical. But, however much this system speaks to the sentiment, preservation is impossible, except in Egypt. Cremation, the prompt destruction of the body by fire, was practiced among the Greeks, ancient Scandinavians, Romans, Celts, Sarmatians, Scythians, Thracians, and ancient Germans and Britons. It is now in vogue among the Hindoos and other Asiatic and aboriginal American peoples. Drying by exposure to sun and dry winds, by hanging in trees, was an ancient custom among some tribes, and is a method in use to-day in some parts of Australia and South America. Chemical disintegration, with corrosive acids or alkalis, is one of the modern propositions, which would not only be offensive, but often efficacious only in part. Sea-burial, an old means, must be rejected, because the sea would soon become too populous with dead; because bodies, carelessly sunk, would be washed ashore to the horror and disease of dwellers thereon, and because fish would probably become less palatable. Petrifaction has lately been tried with incomplete success. Encasing in cement has also been proposed. Any means, however, for the preservation of the dead must have the very cogent objection, that in the course of a few hundred years there would be no room for the living. Since, then, preservation is impossible and undesirable, the question to-day is, Are we to have prompt destruction by fire, or slow by inhumation, or from the more scientific standpoint, which proves the fiery combustion and the combustion in the grave due to the same element, oxygen, Are we to have quick cremation by fire or tardy by burial? That speedy destruction is the more desirable I will endeavor to make evident. It has already been shown that certain of the mephitic gases arising from the decomposition of animal matter, are poisonous, vitiating to a greater or less extent the atmosphere which the living breathe. Soils differ as to the length of time bodies decompose in them. Old world laws recommend graveyards to have a dry, porous, black loamy soil, with a large quantity of superficial vegetable mould, which acts in a manner as an antiseptic, and protest against argillaceous soils as the worst that can be found. But grounds which hasten destruction most release most readily the toxic vapors, and so the best-selected churchyard will exercise an injurious effect upon the atmosphere. In good soils all but the larger bones disappear in twelve years, while in clay it takes fifty years. In Buck's new work on Public Hygiene it is said that Prof. Selmi, of Mantua, has discovered organisms in cemetery airs dangerous to life, and which, injected under the skin of a pigeon, caused death from pyemia. But dead bodies are not only poisonous in this way. There is a two-fold peril. A large majority of our annual deaths are from acute infectious zymotic diseases, and physicians, with that singular inconsistency, which will isolate the contaminated while living, which will burn everything with which they have come in contact, their clothes, utensils, furniture, and sometimes even their house, will yet calmly consign their bodies teeming with the fomites of disease to a churchyard, which, in almost every case, eventually becomes a part of the city which supplies it, which is builded over, and whose soil is sometimes turned up with most disastrous effects. If, in our attempts to eradicate these diseases, we make store-houses of the cemeteries for the use of future generations, what will be our success? We know much of the tenacity of existence disease-germs have even underground. Let me cite a few instances. Trousseau mentions one of a grave-digger who, being called upon many years after the death of a person by small-pox, to re-open the grave, was speedily attacked by the disease. Prof. Bianchi establishes that the plague at Modena, in 1828, was due to the excavations, where three hundred years before victims of the plague had been interred. Mr. Cooper relates a similar outbreak from excavating in an old burying-ground in Eyam, Derbyshire, and further states that the upturning of the soil, where the plague-stricken of 1665 were buried in London, made more virulent the cholera of 1854, a result which had been predicted by Mr. Simon. Dr. Playfair thinks the Roman fever is due to emanation from a soil saturated with organic remains. Eassie states that in 1843 the soil from an old churchyard in Minchinhampton was removed and scattered over neighboring gardens, and was followed by an epidemic, which decimated the village. The outbreak of the plague in Egypt, in 1823, was traced by medical officers, sent out by the French government, to a disused burial-ground at Kelioub, a village near Cairo. At Riom, in Auvergne, the earth of an old cemetery was used to embellish the city, and produced a fatal epidemic, most fatal in the excavated neighborhood. In 1740 a fatal fever in Dublin was distinctly traced to graveyard exhalations. Eassie says that the exhumation of a single corpse buried twelve years engendered disease in a whole convent. Houlier and Femel affirm that the Paris plague of the eighteenth century lingered longest about the Trinity cemetery, and in a report to the French Academy of Medicine it is said that diphtheritic diseases rage near Pere-la-Chaise, Montmartre and Montparnasse, due to the exhalation from those cemeteries. When the yellow fever visited New York in 1822, the mortality was greatest and the disease most persistent in the vicinity of Trinity churchyard. We have learned from the experiments of Tyndall, Bastian and others, that certain organisms may be boiled for hours and may be frozen and still survive to propogate their species. We have heard of the obstinacy with which life lingers in cereals enclosed in Egyptian tombs three thousand years. By what authority then can we affirm that life departs from disease-germs by inhumation ? How dare we preserve vast depots in the south of yellow fever fomites, coffers of Asiatic cholera, and every year accumulate and treasure up small-pox, scarlet fever, whooping-cough, diphtheria and measles ?

In the sixth annual report of the Massachusetts State Board of Health, Dr. Adams, of Pittsfield, from a digest of 171 answers to a circular issued by him to physicians and chemists in this country and Britain, arrives at the following conclusions: That burial in the midst of towns, in contracted grounds, is injurious in proportion to overcrowding of such grounds; that this malign influence is most apparent in epidemics, when mortality is found to be excessive in their vicinity; that extra-mural interment, now generally adopted, prevents injury to public health, and by increasing the number of parks, is a positive sanitary benefit; and that cremation therefore is an innovation not demanded in this country on sanitary grounds. We must say in answer to this, that extra-mural cemeteries, if not established on proper soil, may and do poison the streams which drain them; that in a new country like America, where villages spring magically into cities, an extra-mural cemetery is a provision for the present generation only and is positively certain to be included within the city in a few years; that there is a singular deficiency in state laws, not to be remedied for scores of years, with respect to regulations for the proper location of cemeteries, for the selection of their soils, for the restriction of the number of burials per acre, and for holding them ever after, as is suggested for public parks. We question very much whether there is any large city in the United States, portions of which do not occupy the sites of some of their first cemeteries, and which may not in a century or two build upon their present extra-mural ones.

To those who have so much respect for the dead, and who believes their friend will lie quietly where they have placed them until resurrection, and these credulous ones are legion, it would be an unpleasant reminder of the changes of time, were they permitted to visit the unenduring spots so dear to them a few decades later. It is no uncommon thing in any city for rude workmen to remove the dead utterly regardless of their individuality to a new interment; it is no very uncommon thing for a cemetery in a valley to be exposed and its precious contents devastated by a spring freshet, for such things are related; nor is it uncommon, for I myself have seen it, for cemeteries situated on the banks of changing rivers in alluvial soil, as for instance along the Missouri in the Löess formation, to be gradually undermined and washed away, with coffins protruding from the cliffs, and coffin-boards made into rafts by boys in the stream below. So much for the reverence of people and so much for the reverence of the elements and time.

There are other arguments brought by some against burial which we believe are of less value than those we have mentioned, such as the exposure at funerals to cold and wet and disease, and the economy of cremation. But we hardly credit that the minds of the people will yet permit the use of the ashes of their dead as a fertilizer, nor their consecrated bodies for the manufacture of illuminating gas as the practical Frenchmen suggest. Burial alive will be avoided, although anti-cremationists have taken it upon themselves to doubt whether such a thing has ever occurred, because at Munich and Frankfort the experiments with bell-wires for many years on bodies exposed as long as possible on public biers have failed to resuscitate, as yet, a single case. I need only to refer to a very recent and real case, described in the *London Lancet*, Dec. 8, 1877. It occurred at Naples. Having occasion to open the grave shortly after burial, the result of struggles and anguish was found to have been such as to tear all the clothes and even to fracture some of the bones. The Appeal Court of Naples sentenced the doctor who signed the death certificate and the Mayor who sanctioned the interment to three months imprisonment for "involuntary manslaughter."

It may not be amiss to inquire if our present method of disposing of human bodies is Christian. It is said to have arisen in Rome among the early Christians, but there entombment in catacombs was practiced, not inhumation. Coffins are an Egyptian invention. The Biblical peoples entombed their dead for the Jewish soil with its multitude of natural caves provided for it. These sepulchres were never allowed to be within less than 2,000 cubits of the city walls. Wisdom it seems does not accumulate, but often perishes. Kings might be buried in towns. Bodies were swathed with bandages, saturated with antiseptic spiced oils, and were carried on an open bier to the grave. The mourners bore branches of palm and olive as symbols of joy. How different from what we now consider the Christian method! It seems, however, they had no objection to any other method of disposal, for Saul and his sons were burned. In Amos it is hinted that burning was practiced in time of pestilence. Jacob and Joseph became mummies in Egypt. Inhumation was also often made use of, but coffins were not used and are not even to-day by Christians in the east or Jews or Mohammedans.

One of the grave objections to cremation is undoubtedly the concealment it might sometimes afford poisoners. Early in 1879 the *London Lancet* had one or two editorials, which, though strongly upholding cremation from the sanitary point of view, with equal vigor decried it from the legal. But the writer weakened his statement by confessing that mineral poisons could be discovered sublimated in the gases, or even in the ashes, and by stating that the mineral poisons were rarely used, that vegetable poisons were precisely those to be dreaded, and would be entirely destroyed by incineration of the body. He neglected to state, either through ignorance or purpose, that with the solitary exception of the one alkaloid strychnia, all vegetable poisons decompose with the body, that after death it is extremely rare than any alkaloid can be discovered in the body. Therefore, as to these so much to be dreaded poisons, the alkaloids, the result will be the same whether the body be burned or buried. They will not be discovered. Not one body in 1,000,000, I speak on the authority of a chemist, is disinterred to be examined for suspected poisoning. It is very improbable that anyone to conceal a crime would attempt so difficult a task as destruction of his victim by fire without resort to a crematorium which would be under the direct supervision of an inspector appointed by the city, or under the control of the society owning it. The whole difficulty can be avoided by making the cremation of a human body legal if demanded by two or more persons and the cremation society. Unidentified bodies may be buried, but if cremated because of death from infectious disease, a minute description of the unknown may be preserved. Because cremation is to come into vogue, it is not necessary that public parks be eradicated. On the contrary, the lands occupied and embellished by the crematorium and columbaria would always remain beautiful parks; they would never become nuisances in the midst of a city, and it is unlikely that the artistic structures which would be built thereon—for art too will take a new direction in designing elegant buildings, busts, vases, urns and symbols—would be destroyed or removed, as are old cemeteries.

Popular prejudice is excited against cremation, because popular memory returns to the descriptions of funeral pyres, and of the violence fire does to the dead, in Homer and Virgil, and to the revolting incineration of Shelley on the shores of Tuscany. For this reason it is necessary to state that Venini, Gorini and other Italians, Siemens of Germany, Sir Henry Thompson of England, and others have invented apparatus in which at a temperature of about 2,000° a body may be reduced to ashes in from half an hour to two hours, in some without the flame touching the body, and at an expense for fuel of from seventy-five cents to a dollar and one-half. There is nothing in this repulsive. Between four and six pounds of delicate white fragments of calcined bone remain, which may be inurned or disposed of as friends may please.

The state of cremation to-day is one of progress. Abroad most of the large cities have societies, and several have erected crematories in their public cemeteries. The January number of the *Scientific American* of this year has a cut of the Doric Cremation Temple in the Milan cemetery, illustrative of the Gorini furnace there in use. Cremation has been legalized in Italy since March, 1877, and has been practiced at Milan and Padua. There are several crematories in Germany, and incineration has been performed at Breslau, Dresden, Gotha and other places. The *London Lancet*, of Dec., 1878, reports the Sanitary Council of Munich to have recommended cremation; 1, after battles; 2, during certain epidemics; 3, where the transportation of bodies is difficult; 4, where the soil is unsuited for inhumation. The London Society has probably erected by this time buildings and apparatus in the Great Western Cemetery, this purpose a few years ago having been frustrated by some ecclesiastic authority. The following synopsis of a petition to the home secretary will perhaps better show the attitude of British physicians toward cremation.

"We, the undersigned, members of the British Medical Association, assembled at Cambridge, disapprove of the present custom of burying the dead, and desire to substitute some mode which shall rapidly resolve the body into its component elements by a process which cannot offend the living and may render the remains absolutely innocuous. Until some other mode is devised, we desire to promote that usually known as cremation, etc." T. Spencer Wells,

Aug, 1880. and many others.

In America articles have been written upon the subject by Prof. Fraser, Dr. Adams, Dr. Le Moyne and a number of others. A cremation society was formed in New York some years ago, and within a few days a stock company with $50,000 capital has been established to build a crematorium. A bill has lately been brought before the New York State Legislature providing that cremation be made legal when at the request of three persons. The only crematorium in America is the private one of Dr. Le Moyne, at Washington, Penn., costing $1,500, built in 1877, where last month the tenth body was incinerated. Four or five other cremations have been performed in the United States, and undoubtedly they will grow more numerous as furnaces are established. Some idea, however, of the listlessness of American physicians with regard to this subject may be formed from the following scientific sentence in a letter of Dr. Austin Flint to Dr. Adams, "I can only say that cremation is, in my feeling, at variance with the respect due to the beloved and honored dead." Drs. Ordronaux and Hartshorne and Dr. Herrick of New Orleans, have expressed themselves in favor of cremation when, from lack of room, it shall become necessary in America.

Buffalo supplies twenty-one cemeteries, public and private, many perhaps most of which are within the city limits. But she has had more. Her city and county buildings now rest upon her first tombs. Another old burying ground, once known as the Black Rock cemetery, was situated very near and a little above the North Street Circle. Its site is peopled with the living. The old private cemetery on the corner of Delaware and North streets will not long remain uncovered. There is room for a palatial mansion. One of the oldest churchyards in the city is that on Best street, east of Main, known as the Buffalo cemetery. It covers ten acres, was established in 1836, and is still used. Most of the cholera victims of 1849 were there interred. Probably a fifth of the space is laid out in paths and roads, leaving eight acres for use. According to the English standard of 110 to the acre this should accommodate 880 dead, for perhaps each decade. The number of burials therefore, in order to be sanitarily secure, should be under 4,400 since its existence, but I have reason to believe this number has been greatly exceeded. Of course, no exact statistics can be prepared, for Buffalo as a provincial and somewhat sleepy town has not until within a short time demanded burial permits. But it is certain that several hundred have been buried there yearly for forty-five years, and that thousands were there interred during the cholera epidemic. Whether this cemetery, located in the most elevated portion of the city, contaminates wells in its neighborhood or not, will probably not be determined until health physicians and health boards cease to be political machines, but on the map of this city, recently drawn up by one of the members ot the medical Club, locating the deaths in the scarlatina epidemic of 1873–1874, the greatest mortality was in the immediate neighborhood of this cemetery. However, fifty years hence, I apprehend, when most of the families owning lots in these grounds are dead, the property will revert to the city and be made into a park or more likely be covered with residences. Forest Lawn our largest and most beautiful cemetery of 300 acres has been established for fifteen years. Some 18,000 are already buried there, making an average of sixty interments to the acre. A very few years will make it as populous as it should be in order not to endanger public health. This cemetery will probably always be reserved for a park after it is full and when Buffalo is built all around it. Something should here be said of the soil of our cemeteries. Forest Lawn, which is typical, has from one to three feet of the superficial vegetable mould so desirable on account of its antiseptic properties, but under that it is a compact clay, the very worst soil for inhumation, and below that limestone. It is natural therefore that the rains and graves should drain, not through into the earth, but along the clay slopes into the Scajaquada creek, which flows through Forest Lawn, through the Park and Black Rock, and into the Niagara River. In a recent examination of some ice from this creek by Dr. Davidson of this city, a very large quantity of organic matter was found, showing that the water, which when frozen becomes much purer, is entirely unfit for use, and poisonous. I have no knowledge of the amount or quality of disease prevalent upon its banks. It would be useful to study.

Every city has its shadow. There is a Buffalo over and a Buffalo underground. There is a Buffalo with fine houses and daylight and sunshine and pure air and populous streets. There is a Buffalo with narrower, peopleless streets, with coldness and darkness and silent cells, with still inhabitants, and an atmosphere which is the breath of pestilence. Visitors go there from the brighter city, but return not. The Buffalo above ground grows, but the underground city grows faster. If reverence be really due the dead, let them go not there. Let us lift this shadowy city into the sunshine. Let the elements go, if need be, by fire, purely and speedily into the sunlight whence they came. Perhaps after all I was harsh in saying cremation awaits the evolution of good sense from popular sentiment. It may be that it is a finer sentiment it awaits, the sentiment which prefers the immediate and taintless translation of dead friends into the elements which composed them to the slower combustion of the corrupt and loathsome grave.

